# Editorial: Algal photosynthesis

**DOI:** 10.3389/fmicb.2022.1112301

**Published:** 2023-01-04

**Authors:** Weimin Ma, Lu-Ning Liu, Qiang Wang, Deqiang Duanmu, Bao-Sheng Qiu

**Affiliations:** ^1^College of Life Sciences, Shanghai Normal University, Shanghai, China; ^2^Institute of Systems, Molecular and Integrative Biology, University of Liverpool, Liverpool, United Kingdom; ^3^State Key Laboratory of Crop Stress Adaptation and Improvement, School of Life Sciences, Henan University, Kaifeng, China; ^4^State Key Laboratory of Agricultural Microbiology, College of Life Science and Technology, Shenzhen Institute of Nutrition and Health, Huazhong Agricultural University, Wuhan, China; ^5^Hubei Key Laboratory of Genetic Regulation and Integrative Biology, School of Life Sciences, Central China Normal University, Wuhan, China

**Keywords:** algae, light energy harvesting, photosynthetic membrane complexes, photosynthetic electron transfer, carbon dioxide-concentrating mechanism, carbon fixation, antioxidant mechanism, evolution

Algae are a diverse group of predominantly aquatic photosynthetic organisms, including cyanobacteria, green algae and other eukaryotic algae. They account for more than 50% of the photosynthesis that takes place on Earth. Photosynthetic efficiency is generally higher in algae than in higher plants, because of a wide range of antenna pigments to harvest more solar energy and a variety of carbon dioxide-concentrating systems to increase carbon dioxide (CO_2_) concentration around ribulose-1,5-bisphosphate carboxylase/oxygenase (Rubisco). Apart from their natural role as primary producers in the global carbon cycle, algae also feature in biotechnology for the production of a range of high-value natural products and a sustainable source of protein. This Research Topic aims to gather knowledge on understanding the working mechanisms of efficient photosynthesis in algae and contains 12 papers of which 8 are original research, 3 are reviews or mini-reviews, and one is a perspective.

The oligomeric states of cyanobacterial photosystem I (PSI) are diverse and at least consist of its monomer, dimer, trimer and tetramer. Such diversity is of significant importance for the survival of cyanobacterial cells under changing ecological environments. Chen, Liu, et al. review the implications of structural and oligomeric diversity among cyanobacterial PSI supercomplexes. Through biochemical and biophysical characterization and cryo-EM single-particle analysis, Chen, He, et al. further identified two novel oligomeric states of PSI, hexamer and octamer, from the filamentous cyanobacterium *Anabaena* sp. PCC 7120 grown in a low light environment. Du et al. construct a photo-bio-electrochemical system and in this system, purified reaction center-light harvesting (RC-LH) complex as a mediator can accept the electron from hydroxymethylferrocene (FcMeOH) and transfer to the overlapped fluorine-doped tin oxide (FTO) electrode, being composed of a FTO glass as the front electrode and a Pt-coated FTO glass as the counter electrode. This indicates that purified RC-LH complex can operate in this *in vitro* system. In addition, the activity and stability of PSI are significantly reduced and phototropic growth is significantly attenuated in a *Chlamydomonas reinhardtii* heme oxygenase 1 mutant (*hmox1*) that is deficient in bilin biosynthesis. Zhang et al. reveal the presence of an alternative bilin biosynthetic pathway independent of heme oxygenase 1 in the chloroplast by a *hmox1* suppressor screening in *Chlamydomonas* cells.

Photosynthetic ferredoxin:plastoquinone oxidoreductase (NDH-1) is predominantly, if not totally, located in the thylakoid membrane, accepts electrons from reduced ferredoxin by PSI, and participates in a variety of bio-energetic reactions, including cyclic electron transfer around PSI, CO_2_ acquisition, and cellular respiration. Mi describes the current advances and possible regulatory mechanisms of cyanobacterial NDH-1 in photosynthesis. Translocation of chloroplast-located genes to mitochondria or nucleus is considered to be a safety strategy that impedes mutation of photosynthetic genes and maintains their household function during evolution. Yu et al. propose that the organelle translocation strategy of photosynthetic NDH-1 genes during evolution is necessary to maintain the function of photosynthetic NDH-1 as an important antioxidant mechanism for efficient photosynthesis.

Cyanobacteria use an inorganic carbon-concentrating mechanism (CCM) to increase inorganic carbon concentration around Rubisco for efficient CO_2_ fixation. Tang et al. reveal distinct molecular components and organization of CCM in thermophilic cyanobacteria using the comparative genomic analysis. Their findings provide insights into the CCM components of thermophilic cyanobacteria and fundamental knowledge for further research regarding photosynthetic improvement and biomass yield of thermophilic cyanobacteria with important biotechnological potentials. In addition, through structural analyses and molecular dynamic simulations, Min et al. reveal a previously unrecognized mechanism for the uncommon intermolecular Coenzyme A (CoA) transfer reaction, a key reaction intermediate for carbon fixation. This discovery not only broadens the knowledge on the catalytic mechanisms of CoA transferases, but also contributes to enzyme engineering of the 3-hydroxypropionate cycle for synthesis of high-value chemicals.

During algal evolution, a variety of antioxidant mechanisms are developed to protect algal photosynthesis under harsh environment conditions. Iron-stress-induced protein A (IsiA) is the major chlorophyll-containing protein in iron-starved cyanobacteria, binding up to 50% of the chlorophyll in these cells. Jia et al. describe progress in understanding the regulation and functions of IsiA based on laboratory research using model cyanobacteria. Abscisic acid (ABA) is known as a stress related hormone and plays a critical role in the regulation of various types of stress responses. Yang et al. propose that ABA is synthesized in *Neopyropia yezoensis* possibly *via* the carotenoid, mevalonate (MVA) and 2-C-methyl-D-erythritol 4-phosphate (MEP) pathways and the up-regulation of antioxidase genes under high salinity is mediated by the ABA signaling pathway.

A greater plasticity of metabolic pathways in response to the trophic growth mode is of significant importance for cyanobacterial growth and environmental acclimation. Muth-Pawlak et al. propose the regulatory patterning of carbon metabolism in cyanobacterial cells grown under different trophic modes (including low-carbon autotrophy, carbon-rich autotrophy, photomixotrophy and light-activated heterotrophy) *via* a comparative proteomic strategy. On Earth, far-red light derived photosynthesis occurs in cyanobacteria living in environments where visible light is strongly attenuated. Billi et al. identify the endolithic, extremotolerant cyanobacterium *Chroococcidiopsis* sp. CCMEE 010 capable of far-red light photoacclimation (FaRLiP) with a significantly reduced FaRLiP cluster, which has implications for the possibility of oxygenic photosynthesis on exoplanets.

Collectively, this volume of the Research Topic provides exiting works in the area of the catalytic and antioxidant mechanisms of algal photosynthesis, ranging from the structure, biogenesis, metabolism, signaling, regulation and evolution of photosynthesis in algae to the molecular components and modules of efficient photosynthesis in algae. With the efforts of many researchers worldwide, the frontiers of this topic keep evolving at a rapid pace.

## Author contributions

All authors listed have made a substantial, direct, and intellectual contribution to the work and approved it for publication.

